# Neonatal-onset dilated cardiomyopathy as the initial manifestation of Alström syndrome: a case report

**DOI:** 10.3389/fped.2026.1746048

**Published:** 2026-05-07

**Authors:** Hua Wang, Wandong Ma, Jingshi Chen, Bo Li

**Affiliations:** 1 Department of Pediatric Cardiology, Children’s Hospital of Hebei Province, Shijiazhuang, China; 2 Hebei Clinical Research Center for Children’s Health and Diseases, Shijiazhuang, China; 3 Hebei Provincial Key Laboratory of Pediatric Cardiovascular Medicine, Shijiazhuang, China; 4 Department of Neurosurgery, Hebei General Hospital, Shijiazhuang, China

**Keywords:** Alstrom syndrome, case report, diagnosis, dilated cardiomyopathy, nystagmus

## Abstract

**Background:**

Obesity–retinopathy–diabetes syndrome, also known as Alström syndrome (AS), is an extremely rare autosomal recessive disorder caused by pathogenic variants in the Alström syndrome 1 (*ALMS1*) gene. Its estimated incidence is 1–9 cases per million, with approximately 1,050 cases reported worldwide. AS is characterized by progressive, multisystem dysfunction with age-related and variable clinical features, often leading to multi-organ failure and premature death.

**Case description:**

We report a female infant who initially presented with reversible heart failure in early infancy and subsequently developed nystagmus at six months of age. Genetic testing identified compound heterozygous pathogenic variants in the *ALMS1* gene, confirming the diagnosis of AS.

**Conclusion:**

AS is characterized by multisystem involvement and variable age-related clinical expression. Cardiac dysfunction may precede classic features such as nystagmus or obesity, emphasizing the need for early genetic screening in infants with unexplained cardiomyopathy. Prompt diagnosis facilitates targeted management and improves outcomes.

## Introduction

Alström syndrome (AS) is a rare autosomal recessive disorder caused by biallelic pathogenic variants in the Alström syndrome 1 (*ALMS1*) gene (1). Also known as obesity–retinopathy–diabetes syndrome, it was first described by Alström et al. in 1959, with an estimated incidence of 1–9 per million (1, 2). AS involves multiple organ systems and is characterized by nystagmus, progressive visual and hearing impairment, obesity, diabetes mellitus, renal insufficiency, hypogonadism, and dilated cardiomyopathy (DCM).

AS typically presents in infancy or childhood with obesity, retinitis pigmentosa, sensorineural hearing loss, and type 2 diabetes (3). DCM, however, may precede these hallmark features and can present even in the neonatal period, sometimes as the initial manifestation (3). Such early-onset cases progress rapidly and pose diagnostic challenges, as confirmation relies on genetic testing.

This report describes a neonate with AS initially presenting with DCM and reviews relevant literature to characterize the clinical, genetic, and prognostic landscape of AS in early infancy.

## Case presentation

Written informed consent for publication of this case report was obtained from the legal guardian. Ethical approval was obtained from the hospital's ethics committee (Medical Ethics No. 202136). The report follows the CARE guidelines (4).

### Patient information

A 25-day-old female infant was admitted with an 8-day history of cough and 3 days of labored breathing, with signs of cardiac dysfunction. She was the second child of a third pregnancy, delivered by cesarean section at 38 + 5 weeks (birth weight: 3750 g). Prenatal and family history were unremarkable.

### Clinical findings

Laboratory investigations revealed mild anemia (hemoglobin 102 g/L, reference range: 110–160 g/L) and elevated cardiac biomarkers, including troponin-1 (1.299 μg/L, reference <0.058 μg/L) and B-type natriuretic peptide (BNP) (5,315 pg/mL, reference <100 pg/mL). Coagulation studies showed prolonged activated partial thromboplastin time (APTT) (36.7 s, reference range: 23.3–32.5 s) and elevated D-dimer (0.75 mg/L, reference <0.05 mg/L). Liver and renal function tests were normal, except for mildly elevated creatine kinase MB isoenzyme mass (CK-MB) (10.72 μg/L, reference <5 μg/L) and lactate dehydrogenase (343 U/L, reference range: 110–160 U/L). Cytokine analysis demonstrated increased levels of interleukin (IL)-4 (6.20 pg/mL, reference <3.0 pg/L), IL-6 (71.5 pg/mL, reference <20.0 pg/L), IL-10 (6.88 pg/mL, reference <5.9 pg/L), and TNF-α (7.4 pg/mL, reference <5.5 pg/L).

Chest CT revealed bronchopneumonia and a slightly enlarged cardiac silhouette. Echocardiography showed left atrial (LA) and left ventricular (LV) enlargement [LA diameter: 22 mm [normal upper limit: 11.6 mm], LV end-diastolic diameter [LVDD]: 31 mm [normal upper limit: 25.5 mm]], with an ejection fraction (EF) of 39% (reference range: 65%–75%) and fractional shortening (FS) of 18% (reference range: 40%–45%). Additional findings included apical trabecular thickening, moderate mitral regurgitation, and mild tricuspid regurgitation. Electrocardiogram (ECG) showed sinus tachycardia (155 bpm, normal: 120–140 bpm) with T-wave abnormalities. Other metabolic and endocrine parameters were normal.

### Diagnosis and management

The diagnoses included dilated cardiomyopathy, moderate cardiac dysfunction (modified ROSS score: 7), pneumonia, and myocardial injury. The patient received antibacterial therapy (amoxicillin–clavulanate), inotropes and diuretics (digoxin [6.15 μg/kg/d], milrinone [loading dose 30 μg/kg, maintenance dose 0.41 μg/kg/min], spironolactone [2 mg/kg/d], hydrochlorothiazide [2 mg/kg/d]), metabolic and cardioprotective agents [creatine phosphate [0.25 g/d], fructose diphosphate sodium oral solution [5 mL twice daily], captopril [initial dose 0.38 mg/kg/d, gradually increased to 0.86 mg/kg/d]], and anti-inflammatory and immunomodulatory therapy (intravenous human immunoglobulin and prednisone), and adjunctive levocarnitine oral solution (5 mL once daily).

### Follow-up and outcome

At discharge, echocardiography showed partial improvement (LA diameter 13 mm, LVDD 28 mm, EF 43% and FS 20%), and BNP had decreased to 317 pg/mL. Post-discharge, the infant was continued on oral digoxin, prednisone acetate, furosemide, spironolactone, captopril, potassium chloride granules, and calcium supplements and attended regular outpatient follow-up. By four months, the clinical symptoms had resolved, and echocardiography showed normal cardiac function. During follow-up, the infant has not exhibited polydactyly, intellectual disability, or hypogonadism. At six months of age, she developed binocular nystagmus and limb tremors. She underwent fundus examination as well as magnetic resonance imaging (MRI) of the head and orbits, all of which revealed normal findings. Genetic evaluation for Alstrom syndrome was performed after obtaining informed consent from her parents.

### Genetic findings

**Figure 1:**
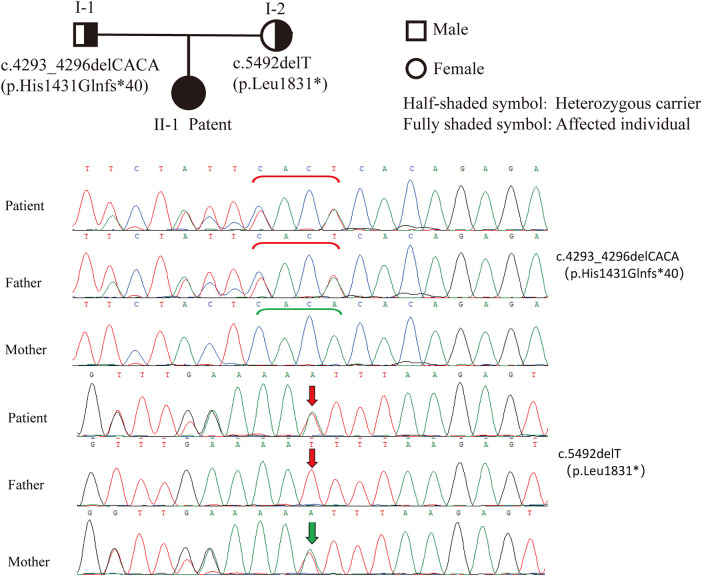
*ALMS1* gene sequencing results of the patient and parents. The proband carries a heterozygous c.4293_4296delCACA (p.His1431Glnfs*40) variant, inherited from the father, and a heterozygous c.5492delT (p.Leu1831*) variant inherited from the mother.

Whole-exome sequencing revealed compound heterozygous pathogenic variants in the *ALMS1* gene: c.4293_4296delCACA and c.5492delT, both located in exon 8. These frameshift mutations resulted in premature termination codons, leading to truncated protein products (Figure 1). The c.4293_4296delCACA variant is a known pathogenic mutation listed in the HGMD database [PubMed: 2459103], while c.5492delT is a novel variant. Parental testing confirmed heterozygous carrier status, establishing the diagnosis of autosomal recessive Alström syndrome.

## Discussion

AS is a rare autosomal recessive monogenic disorder involving multiple organs, with marked phenotypic heterogeneity across individuals and age groups. Characteristic features include obesity, progressive cone–rod dystrophy leading to blindness, sensorineural hearing loss, hypertriglyceridemia, short stature, insulin resistance, childhood-onset type 2 diabetes, hypertension, and multi-organ dysfunction (5). Diagnosis is based on age-stratified major and minor criteria (Table 1). Major criteria include identification of pathogenic *ALMS1* variants, family history, or characteristic visual impairment, while minor criteria encompass obesity, insulin resistance or diabetes, DCM, and hearing loss (6). Early signs such as nystagmus and photophobia may appear at birth, whereas hypertension and renal failure typically develop later. Because of its nonspecific features, AS is frequently underdiagnosed in early life; hence, genetic testing should be considered in infants with unexplained DCM, nystagmus, or sensorineural hearing loss (6).

**Table 1:** Age-based diagnostic criteria for alstrom syndrome.

Age Range	Diagnostic criteria		Clinical Diagnosis
	Major	Minor	
Birth–2 years	Pathogenic variants in *ALMS1* or family history of Alstrom syndrome; Nystagmus/photophobia/visual impairment; Pediatric cardiomyopathy	Obesity; Sensorineural hearing loss	2 major criteria or 1 major criterion+2 minor criteria
3–14 years	Pathogenic variants in *ALMS1* or family history of Alstrom syndrome; Nystagmus/photophobia/visual impairment; Pediatric cardiomyopathy	Sensorineural hearing loss; Obesity and/or its complications (e.g., insulin resistance, type 2 diabetes, hepatic steatosis, hypertriglyceridemia); Restrictive cardiomyopathy; Renal dysfunction	2 major criteria or 1 major criterion+3 minor criteria
15 years–Adult	Pathogenic variants in *ALMS1* or family history of Alstrom syndrome; Vision (infantile/childhood nystagmus, visual impairment, legal blindness, cone-rod dystrophy)	Sensorineural hearing loss; Restrictive cardiomyopathy or history of pediatric cardiomyopathy; Obesity and/or its complications (e.g., insulin resistance, type 2 diabetes, hepatic steatosis, hypertriglyceridemia); Restrictive cardiomyopathy; CKD ≥ stage III	2 major criteria +2 minor criteria or 1 major criterion +4 minor criteria

*ALMS1*, gene symbol for Alstrom syndrome 1; CKD, chronic kidney disease.

This case describes a neonate with DCM and heart failure as the initial presentation of AS, an exceptionally rare occurrence. *ALMS1*, located on chromosome 2p13, spans approximately 224 kb with 23 exons encoding a 4,169–amino acid protein (nM_015120.2) (7). The *ALMS1* protein localizes to centrosomes and basal bodies of ciliated cells in multiple organs, including the retina, heart, kidneys, and endocrine tissues. To date, 467 *ALMS1* mutations have been identified, mainly nonsense or frameshift variants (3, 7). Most occur in exons 8, 10, and 16, accounting for over 90% of reported mutations in European cohorts (8).

In this patient, compound heterozygous variants, c.4293_4296delCACA and c.5492delT, were detected in exon 8 of chromosome 2. The c.4293_4296delCACA variant has been associated with DCM, retinopathy, and obesity (9), while c.5492delT is a frameshift mutation predicted to cause premature termination or nonsense-mediated decay. Both were classified as pathogenic under ACMG guidelines (10).

*ALMS1* localizes to the centrosome and basal body of cilia (11), playing a critical role in ciliary formation and maintenance; therefore, AS is classified as a ciliopathy (5). Primary cilia regulate signaling pathways essential for cardiac morphogenesis (12). Loss of *ALMS1* disrupts centrosomal cohesion and ciliary stability, leading to developmental and functional cardiac abnormalities. Mutations in *ALMS1* can result in various types of cardiomyopathies other than DCM. In approximately 20% of AS cases, restrictive cardiomyopathy with fibrosis and pulmonary hypertension develops during adolescence or adulthood. As reported by Dedeoglu et al., patients harboring the c.7911dupC (p.Asn2638Glnfs*24) mutation may present with restrictive cardiomyopathy, while another case exhibited prominent left ventricular trabeculations with deep intertrabecular recesses perfused from the ventricular cavity (13).

According to Marshall et al. (14), in patients with AS, heart failure accounts for ∼90% of deaths in childhood, whereas in adulthood, the combined proportion of deaths attributable to heart failure or renal failure is ∼60%, suggesting a reduced contribution of heart failure to mortality in adulthood. Approximately 40% of affected infants develop transient but severe cardiomyopathy within the first month of life (6). AS-related cardiomyopathy includes acute infantile and late-onset types. The infantile form manifests within three months of birth and may improve with standard anti-heart failure therapy, though residual myocardial damage can predispose to recurrence. Even patients without overt infantile cardiomyopathy may have subclinical myocardial involvement, with a 25% risk of stress-induced heart failure (5). The most severe form, termed mitotic cardiomyopathy, is characterized by persistent cardiomyocyte mitosis driven by aberrant Wnt/*β*-catenin signaling, activation of transcription factors TCF/LEF, and cell-cycle gene overexpression (9). Late-onset cases typically demonstrate myocardial fibrosis (5), while adolescents and adults may develop restrictive cardiomyopathy with pulmonary fibrosis and pulmonary hypertension (14). ALMS1.

Management of cardiovascular complications includes several aspects (15, 16): (1) Basic therapy: comprehensive cardiac evaluation, regular follow-up, and standard pharmacological management with angiotensin-converting enzyme inhibitors, β-blockers, and aldosterone receptor antagonists. Ivabradine may be used in adolescents and adults with resting heart rates >72 beats/min. Cardiac resynchronization therapy is considered for patients with QRS duration >130 ms and persistent symptoms. Diuretics help control chronic volume overload. (2) Prevention of sudden cardiac death: Implantable cardioverter-defibrillators are indicated for survivors of cardiac arrest, patients with hemodynamically unstable ventricular arrhythmias, or those with an ejection fraction <35% and expected survival >1 year. (3) End-stage heart failure therapy: refractory cases may require temporary mechanical circulatory support or heart transplantation. (4) Cardiovascular risk management: older children and adults with AS frequently exhibit insulin resistance and features of metabolic syndrome, increasing their risk of subclinical ischemic cardiovascular disease.

AS carries a poor prognosis, with some patients dying in infancy due to heart failure or pulmonary edema. Adults typically succumb to multi-organ failure, and the average life expectancy is under 50 years. At present, no curative therapy exists; management remains largely supportive and symptom-directed. Lifestyle interventions, such as visual aids, physical activity, and dietary control, can help mitigate metabolic complications (6). Metformin and other insulin sensitizers improve insulin resistance, while insulin replacement therapy may be used when indicated. PBI-4050 (sodium 3-pentylphenylacetate) has shown anti-inflammatory and antifibrotic activity in preclinical studies and is under clinical evaluation (17, 18). Emerging strategies, including gene and stem cell therapies, hold promise; preclinical studies suggest that gene replacement therapy can restore ciliary function in AS models (11).

## Conclusion

Early diagnosis of AS primarily relies on detailed phenotypic assessment and *ALMS1* sequencing. The absence of characteristic early features often delays recognition. In this patient, recovery from heart failure followed by the onset of nystagmus demonstrates the progressive nature of disease expression, indicating that cardiac dysfunction may precede hallmark features such as nystagmus and obesity. AS should be considered in infants with unexplained DCM or multi-organ involvement, and early genetic testing is crucial for confirmation. This case also reports a novel *ALMS1* mutation, expanding the known mutational spectrum of the gene.

## Data Availability

The raw data supporting the conclusions of this article will be made available by the authors, without undue reservation.
